# Impact of COVID-19 on an Urban Refugee Population

**DOI:** 10.1089/heq.2020.0148

**Published:** 2021-10-06

**Authors:** Ila Gautham, Sophie Albert, Aisha Koroma, Sophia Banu

**Affiliations:** ^1^ Baylor College of Medicine, School of Medicine, Houston, TX, USA.; ^2^ Alliance for Multicultural Community Services, Houston, Texas, USA.; ^3^ Menninger Department of Psychiatry and Behavioral Sciences, Baylor College of Medicine, Houston, Texas, USA.; ^4^ Clinic for International Trauma Survivors, Houston, Texas, USA.

**Keywords:** COVID-19, refugee, pandemic, health disparities

## Abstract

**Purpose:** The COVID-19 pandemic has brought to light many systemic inequities in health care delivery. As medical communities work to address the disproportionate effects of COVID-19 on vulnerable populations, it is crucial to include refugees in the public health response. Language barriers, poor health literacy, and low socioeconomic status render refugee populations highly susceptible to negative outcomes from the COVID-19 pandemic. To better understand the refugee experience with COVID-19, we constructed and administered a survey among refugee populations in Houston, Texas.

**Methods:** Our 49-question cross-sectional survey was administered to 44 participants in Arabic, Burmese, Dari, English, Kiswahili, Nepali, Spanish, or Urdu with the use of refugee resettlement case managers acting as translators. The survey encompassed three domains, including a general knowledge assessment of COVID-19, subjective experiences with COVID-19, and risk communication practices within refugee populations.

**Results:** The majority of refugees surveyed admitted to worrying about the effects of COVID-19 on their community (88.6%). The negative consequences of the COVID-19 pandemic included financial adversity (65.1%) and significant disruption of children's education (62.8%). Although 50.0% of participants self-reported proficiency in English, translation services were used with 75.0% of participants to ensure full comprehension.

**Conclusions:** The implications of our findings suggest that local refugee populations require heightened support during the COVID-19 pandemic. Tailored interventions should encompass comprehensive translation and interpretation services, financial assistance, and academic interventions for refugee youth.

## Introduction

As the COVID-19 pandemic continues to threaten lives and health care systems globally, national public health measures have been implemented to suppress community transmission of this novel coronavirus. A combination of rigid government regulations and educational outreach efforts has limited social interactions and encouraged individual protective measures. Literature suggests that in the midst of similar past public health crises, the needs of vulnerable populations such as refugees have been neglected or overlooked.^1^ In the United States, the pandemic has starkly exposed pre-existing social and economic disparities and exaggerated their negative effects on health outcomes.^2^

The United Nations defines a refugee as any individual who has fled war, violence, conflict, or persecution and has crossed an international border to find safety in another country.^3^ The COVID-19 pandemic threatens the health and well-being of refugees resettled in the United States by exacerbating the existing social disparities that these populations face. Refugee populations are more likely to reside in crowded living areas, more likely to be of low socioeconomic status, and more likely to work in service industries with high exposure to COVID-19.^4^ COVID-19-related mandates to close down the workplace threaten the economic stability for many refugee families due to loss of employment and income. Due to a lack of steady income or reliable public health information, many refugee families may not have adequate supplies such as soap, hand sanitizer, thermometer, or personal protective equipment. Refugee populations may also lack reliable transportation, housing, health insurance, or access to primary care physicians and medical services.^4^

Refugee populations face increased health risks due to the COVID-19 outbreak as the ability to disseminate reliable information regarding COVID-19 symptoms, prevention, and treatment is compromised. A combination of language barriers, low health literacy rates, and propagation of misinformation through variable knowledge sources poses a significant barrier to health care.^5^ Furthermore, recent policy changes related to public benefits for immigrants further marginalize refugee communities.^6^ Refugee populations may be fearful of utilizing public health care resources in case it affects their future immigration or visa status. Additionally, those within the refugee community may be wary of seeking treatment for COVID-19 should it put any family or friends who remain undocumented at risk of deportation or detention.

The COVID-19 crisis additionally threatens the educational progress of refugee children. Parental language barriers may prevent refugee students from receiving an equal caliber of home learning that occurs in their American classmates' households.^7,8^ A lack of technological resources or reliable internet may prevent the use of online classroom platforms and access to competent teachers. The COVID-19 outbreak may therefore cause significant disruption in the education of refugee children, such that they fall significantly behind their American classmates once school resumes.

The unique challenges that refugee communities face in the midst of global health crises require specifically targeted culturally sensitive interventions. As a large metropolitan city with a significant influx of refugees, the city of Houston must work to integrate the needs of refugees into its COVID-19 response.^9^ However, there are no studies specifically from Houston addressing the impact of the pandemic on refugees, their challenges, and their needs. Such information is required to plan and implement effective response efforts. Therefore, we performed this study with the following goals: to identify knowledge deficits, resource deficits, attitudes toward the pandemic, barriers to health care, disruption in youth education, and economic impacts of COVID-19 on the local refugee population.

## Methods

This study was conducted by a multidisciplinary group with the Baylor College of Medicine Department of Psychiatry and The Alliance (formerly known as Alliance for Multicultural Community Services) in collaboration with a risk communication-based working group funded by CONVERGE, a national science foundation-funded initiative of the Natural Hazards Center at the University of Colorado Boulder. This working group initiative includes the Living Hope Wheelchair Association and the Houston Immigration Legal Services Collaborative.

We developed a survey to address the goals of our study. The initial draft survey was revised with input from members of the working group. The final survey consisted of 49 questions organized into three domains: (1) clinically relevant questions to assess the accuracy of general knowledge, (2) subjective COVID-19 perceptions and experiences, and (3) prevailing risk communication practices within refugee communities. The first domain is modeled after an existing survey addressing knowledge and perceptions of COVID-19 in the general public, it was simplified and tailored for refugee populations with author's permission.^10^ Subsequent domains were devised with input from experienced members of the CONVERGE working group. The options for survey question responses were yes or no, a Likert scale graded from 1–5 (1=strongly disagree, 2=disagree, 3=neutral, 4=agree, and 5=strongly agree), and open-ended comments.

The survey was administered to a convenience sample of 44 refugees associated with the Refugee Programs Department at the Alliance for Multicultural Community Services in Houston, Texas. Refugee resettlement case managers who are fluent in their clients' languages of Arabic, Burmese, Dari, Kiswahili, Nepali, Spanish, or Urdu offered the survey to a minimum of five clients each. These languages were selected as they represent a significant component of refugee arrivals to Houston in recent years. All individuals aged 18 years or older were eligible, irrespective of gender or sex, if they had been granted refugee status by the US government and had moved to the United States within the preceding 5 years, with permanent residence in southwest Houston. Participants also required access to a reliable phone line and were required to provide verbal consent before beginning the study procedure. Only one survey was administered per family, and each participant was provided with an incentive of a $15 gift card to a local grocery store, purchased with funds supplied by CONVERGE. At the end of the survey, each participant was offered a debriefing session, where they could use a translator to ask any questions regarding the COVID-19 pandemic in a nonjudgmental environment.

Current data at the Alliance Refugee Programs Department show that a significant portion of affiliated refugees who have resettled locally are illiterate, therefore this survey was administered verbally in a semistructured interview format. Each survey was administered over the phone or in person by a study investigator, with case managers acting as translators for non-English-speaking participants. Study investigators ensured standardization in survey administration and documented all answers on the free online platform, Google Forms. Case managers have a close prior relationship with their respective clients, therefore participants were more likely to answer their phone calls, trust their case manager as an unbiased translator, and feel comfortable disclosing personal beliefs to their case manager over the phone.

Survey data were analyzed based on response type. Continuous data responses regarding perception and personal experiences were divided into affirmatory responses (agree/strongly agree), neutral responses, and negative responses (disagree/strongly disagree), with medians reported for each variable.

Analysis is ongoing for qualitative risk communication-based data and will be reported upon completion among all members of the CONVERGE working group. For categorical data, such as symptoms and prevention methods of COVID-19, responses are being manually dichotomized into correct and incorrect responses, in line with current CDC public health protocols.

This study was performed with IRB approval of the Baylor College of Medicine Biomedical Research and Assurance Information Network (BRAIN), which oversees human subject research. All research was conducted independently among working group members, and no identifying data gathered through the refugee research initiative were shared with other members of the working group.

## Results

We interviewed 44 subjects between July 15, 2020, and April 4, 2021. These individuals represented 94% of the 47 individuals we approached, who consented and were available for an interview. Of the interviews conducted, 75% required the use of a case manager as a translator.

The mean (standard deviation) reported age of the participants was 34.2 (11.3) years and 55% were female.

Of the subjects, 20% reported having had a positive test for COVID-19 or having a close family member test positive before or at the time of the interview.

Among the subjects, the distribution of the primary language in which the interview was conducted was as follows: English 25.0%, Spanish 18.2%, Arabic 18.2%, Kiswahili 16.0%, Nepali 11.4%, Burmese 4.5%, Dari 4.5%, and Urdu 2.3%. The distribution of the primary language spoken at home by the subjects was as follows: Arabic 22.7%, Spanish 20.5%, Kiswahili 18.2%, Nepali 1.4%, Kachin 9.1%, Farsi 4.5%, Pashto 4.5%, Burmese 2.3%, Dari 2.3%, English 2.3%, and Urdu 2.3%. A breakdown of demographic information for all participants is shown in Table 1.

**Table 1.** Participant Demographic Information

Demographic categories	No. of participants	% Of total sample^a^
Gender		
Male	20	54.5
Female	24	45.5
Age^b^ (years)		
18–29	18	42.3
30–49	19	45.2
50+	5	11.9
Employment		
Currently employed	14	31.8
Currently not employed	30	68.2
Primary language spoken at home		
Arabic	10	22.8
Burmese	1	2.3
Dari	1	2.3
English	1	2.3
Farsi	2	4.5
Kachin	4	9.1
Kiswahili	8	18.2
Nepali	5	11.4
Pashto	2	4.5
Spanish	9	20.5
Urdu	1	2.3
English proficiency		
Proficient	22	50.0
Not proficient	22	50.0

^a^ Cumulative percentages may not add up exactly to 100% per section due to rounding of data.

^b^ Two participants declined to provide their age.

Personal proficiency in English was self-reported by 50.0% of subjects, and proficiency in English of one or more persons at home was reported by 84.1% of subjects. The availability of emergency information in participants' respective primary languages was reported by 65.9%. Of these, 75.9% stated that the information was timely and 96.6% stated that the information was useful. When asked who participants primarily communicate with for any questions related to disasters or the COVID-19 pandemic in Houston, 45.5% reported that they asked family members or friends, 34.1% asked their contact at The Alliance, 9.1% asked either family or their contact at The Alliance, and 11.4% accessed the news through either television or the internet.

Of the subjects, 93.2% reported having WiFi at home and 86.4% reported having a smartphone with internet access; 66.0% reported having a laptop or tablet with internet access. Among the subjects with children or grandchildren, 73.0% reported that the children had separate devices to complete schoolwork during the pandemic. Of those with children or grandchildren, 75.8% reported that the children had received a laptop, tablet, or electronic device from their school or different organization due to the COVID-19 pandemic.

A job was currently held by 31.8% of the subjects. Of those with jobs, 57.1% were able to take time off from work during the COVID-19 pandemic. Of those subjects without a job, 33.3% reported losing their job due to the pandemic. Of note, 65.8% of participants agreed or strongly agreed that the COVID-19 outbreak has negatively impacted their financial situation. Expanding on this, 61.5% of participants agreed or strongly agreed that the pandemic has impacted their ability to pay rent or provide housing, while 41.0% agreed or strongly agreed that it has impacted their ability to purchase food. Full Likert scale responses to specific questions about the subjects' understanding of the pandemic, preventive measures, and personal experiences are shown in Table 2.

**Table 2.** Subjects' Perceptions and Experiences of the Pandemic and Preventive Measures

Question/item choices	Percentage			Median of Likert scale response
	Disagree or strongly disagree (%)	Neutral (%)	Agree or strongly agree (%)	
I have a good understanding of the COVID-19 pandemic	4.5	6.8	88.6	4
I understand the stay-in-place restrictions and/or mask mandates	2.3	2.3	95.5	5
It is easy for me to remain six feet away from nonfamily members during daily activities	20.5	6.8	72.7	4
I am worried about the impact of the pandemic on my community	6.8	4.5	88.6	5
I am worried about myself or my family members getting infected or reinfected with COVID-19	7.0	9.3	83.7	5
I am worried about myself or my family members being detained or deported for seeking out covid-19 resources	68.2	4.5	27.2	2
The COVID-19 outbreak has had a significant impact on my physical health and/or stress levels	13.6	11.4	75.0	4
The COVID-19 pandemic has had a negative impact on my children's or grandchildren's education	16.3	20.9	62.8	4
I feel comfortable educating my children or grandchildren at home in English	34.9	23.3	41.9	3
The COVID-19 outbreak has negatively impacted my financial situation	23.3	11.6	65.1	4
I feel well informed about benefits applicable to me related to unemployment and stimulus checks, etc.	9.1	22.7	68.2	4
The COVID-19 pandemic has impacted my ability to pay rent or provide housing	29.5	9.1	61.4	4
The COVID-19 pandemic has had a negative impact on my ability to purchase or obtain food for my family	29.5	29.5	40.9	3
I have access to adequate cleaning supplies	16.3	9.3	74.4	4
I have access to personal protective equipment	15.9	6.8	77.3	4
I have access to a doctor or other health professional in case of illness	18.2	9.1	72.7	4
I have access to transportation in case of illness	9.1	15.9	75.0	4

Cumulative percentages may not add up exactly to 100% per row due to rounding of data.

## Discussion

The existing vulnerabilities of refugee populations in the United States are further complicated by the unique circumstances of the COVID-19 pandemic. In addition to the financial and educational implications of COVID-19 restrictions, the general well-being of refugee communities must be prioritized in response efforts.

In this study, we found that 88.6% of refugees reported worrying about the impact of the COVID-19 pandemic on their community. Additionally, 83.7% were worried about themselves or a family member getting infected or reinfected with COVID-19. It is widely proven that many refugees experience significant stress from being displaced and experiencing adverse circumstances.^11^ Our findings suggest that the pandemic is likely adding to the mental distress of refugee populations.

Two-thirds of participants also reported adverse financial effects from the COVID-19 pandemic, including reduced ability to pay rent or afford housing (experienced by over 60% of subjects) and reduced ability to purchase food (experienced by 40.9% of subjects). A possible contributor to this was pandemic-related job loss (experienced by almost 33.3% of participants who are currently unemployed). As refugee families work to reestablish themselves in the United States, any financial setback can have drastic long-term implications. While the 31.8% of subjects currently employed may face fewer financial challenges than those who are unemployed, they faced other challenges such as increased risk of COVID-19 infections in the workplace. For example, refugees are more likely than US-born workers to work in low-income fields with higher exposure to COVID-19. Refugees who are in a low-income bracket additionally have an increased likelihood of using crowded public transportation, putting them at greater risk of COVID-19 transmission.^12^

While general anxiety and financial stresses from COVID-19 are widely experienced by the general public, a unique challenge faced by refugees is the language barrier. Not only does a language barrier impair access to care but it also impairs awareness of emerging new information about COVID-19. Half of all participants were not proficient in English, and one participant was quoted saying “I don't understand the whole thing at all because I don't know how to read.” Although 65.9% of all participants could access COVID-19-related information in their primary language, 45.5% of participants stated that they exclusively asked family or friends for information. Reliance on family or friends for information is common in refugee communities, which tend to be tight-knit and whose collectivist mentality offers strong social support.^13^ However, such reliance also puts community members at risk of misinformation.

Another consequence of language barriers during the COVID-19 pandemic is the disruption of refugee youth education. Refugee children already must integrate into new schools while enduring the trauma of fleeing their home countries. COVID-19 risks further disruption of refugee education due to stay-at-home orders and transition of teaching to online mediums. During this crucial developmental period, at-home education requires both electronic resources and parental engagement with younger children. Among our participants, 75.8% had received an electronic device for their children's schoolwork. Even with electronic devices, the usage of unfamiliar educational software programs may be a complex challenge for refugee parents and children. Additionally, only half of the participants with children felt comfortable helping their child with schoolwork in English. The inability to seek help at home puts refugee children at significant risk of falling behind their peers with English-speaking families. Overall, 62.8% of participants with children experienced a negative impact on their children's education.

There were several limitations to this study. Primarily, this survey was not validated before admission. As the survey was executed in eight different languages, despite our attempts to standardize verbal administration, it is possible that participants varied in their interpretation of the questions. Such challenges are intrinsic to the nature of research with refugee populations. However, our findings do provide useful new information in the absence of prior data on this topic. Our study is also subject to selection bias because we only accessed participants with a functioning phone line. This study therefore excluded a subset of refugees who are at increased vulnerability during the COVID-19 pandemic as they are disconnected from all information. Additionally, this study was conducted through The Alliance, so all participants surveyed were already in touch with a support service through their resettlement agencies. This study therefore excluded refugees without a local support system, such as secondary migrants to Houston, who could be facing increased isolation during this time.

Based on our results, we propose that key refugee-inclusive interventions during the COVID-19 pandemic must include financial assistance and identification of refugee youth who risk falling behind in school. It is also essential to have up-to-date demographic data on US refugee communities to allow adequate translation and dissemination of verified COVID-19 resources. Furthermore, this information must be disseminated verbally to ensure that nonliterate community members are included. In Houston, local resettlement agencies such as The Alliance have proven to be essential in the COVID-19 response for refugee communities. Participants admitted to relying on The Alliance for support; 34.1% reported getting all pandemic-related information from The Alliance and an additional 9.1% exclusively asked either The Alliance or family members. The role of personal case managers who are able to speak participants' native language is a crucial method of disseminating information such as financial wellness, job opportunities, health care, and education assistance. While this infrastructure is sustainable at a local level, national measures should aim to include refugee populations in all public education efforts, especially as the development and distribution of a vaccine progress. Public health professionals should work closely with refugee service providers to build capacity and ensure collaboration in crises.

Future studies in this population should work to target a larger more diverse sample of refugees and address more vulnerable subsets of refugees who do not have phone services or connections with a local resettlement service. Rather than considering all refugees as one homogeneous entity, further studies should also identify specific needs of individual ethnic groups within refugee communities. Future investigations should also evaluate pandemic-specific effects on health outcomes such as preventive care, emergency care, and conditions unrelated to COVID-19. Last, refugee youth require targeted research, especially a long-term follow-up on the impacts of the COVID-19 pandemic.

## Conclusions

In conclusion, we found that a high proportion of refugees in the Houston area have experienced significant financial adversity, mental stress, communication barriers, and disruption of children's education during the COVID-19 pandemic. These findings enable us to provide customized and specific interventions to improve refugee health and wellness.

## Abbreviation Used

BRAIN: Biomedical Research and Assurance Information Network
